# “This Is Something That Changed My Life”: A Qualitative Study of Patients' Experiences in a Clinical Trial of Ketamine Treatment for Alcohol Use Disorders

**DOI:** 10.3389/fpsyt.2021.695335

**Published:** 2021-08-16

**Authors:** O. Merve Mollaahmetoglu, Johanna Keeler, Katherine J. Ashbullby, Eirini Ketzitzidou-Argyri, Meryem Grabski, Celia J. A. Morgan

**Affiliations:** ^1^Psychology Department, Psychopharmacology and Addiction Research Centre, University of Exeter, Exeter, United Kingdom; ^2^Department of Clinical, Educational and Health Psychology, Clinical Psychopharmacology Unit, University College London, London, United Kingdom

**Keywords:** alcohol use disorder, ketamine, psychedelics, dissociation, thematic analysis, qualitative study, clinical trial

## Abstract

**Background:** The therapeutic benefits of ketamine have been demonstrated for a variety of psychiatric disorders. However, the role of ketamine induced psychoactive experiences in mediating the therapeutic effects is unclear. Despite the growing quantitative research on the efficacy of ketamine treatment, very few studies examined participant experiences of ketamine infusions in a treatment setting.

**Aims:** The current study aimed to examine participant experiences of ketamine infusions and how these relate to therapeutic mechanisms in a clinical trial setting.

**Methods:** We conducted semi-structured interviews with 12 participants who received up to three ketamine infusions (0.8 mg/kg) as part of a Phase II double blind, randomised controlled trial. The interviews explored participants' acute experiences of ketamine infusions, experiences of psychotherapy/education, and the lasting effects of the trial. The interviews were transcribed verbatim and analysed using thematic analysis.

**Results:** Six key themes were identified. (1) Participants reported multifaceted motivations for trial participation. (2) The set and setting was found to be influential in determining acute ketamine experiences. The acute ketamine experiences included: (3) the inherent contradictions of the experience (e.g., dissociation vs feelings of connection), (4) rapidly fluctuating and changing experiences, (5) meaningful, mystical and spiritual experiences. Finally, the final theme (6) relates to the transformational effects of the infusions and the trial.

**Conclusion:** Provided in a supportive and professional environment, ketamine treatment led to a significant change in relationship with alcohol. Ketamine induced ego dissolution and dissociation were reported to be related to the transformational effects on relationship with alcohol. The extent to which the acute psychoactive effects of ketamine mediate therapeutic effects on drinking outcomes remain to be investigated in the trial data. The acute effects of ketamine reported by our participants transcend its traditional conceptualisation as a “dissociative anaesthetic”; therefore, we suggest the development or use of new measures alongside ketamine infusions to fully capture the spectrum of these effects which may be crucial in its therapeutic and transformative effects.

## Introduction

Ketamine is an N-methyl-d-aspartate (NMDA) receptor antagonist, which produces powerful dissociative effects. Ketamine was initially developed in the 1960s as an anaesthetic drug (1), but there has been a recent escalation in interest for its use in treating psychiatric disorders. Ketamine has been characterised as a “dissociative anaesthetic” due to strong sensory dissociation associated with it (1) [for a review of terminology referring to similar classes of drugs including “psychedelics,” “hallucinogens,” “entheogens,” “psychotomimetics” see (2)]. At a sub-anaesthetic dose, ketamine has been shown to display rapid antidepressant effects (3–5) and there is further evidence demonstrating its therapeutic benefits for a variety of psychiatric disorders such as unipolar/bipolar depression, post-traumatic stress disorder, generalised anxiety disorders, obsessive compulsive disorder, and substance use disorders (6). A small number of studies demonstrate the therapeutic effects of ketamine on drug abstinence, drug use, craving and withdrawal [see (7) for a review], and most recently alcohol use disorder (8, 9). Although the safety and efficacy of ketamine has been well-established, few studies thus far have looked at the phenomenological experiences of people who have been given the drug therapeutically and how these relate to mechanisms of therapeutic benefits (10, 11).

Ketamine acutely can induce mystical and psychedelic effects [for a full review of mechanisms of action see (12)]. Whilst these have been thought of as adverse effects in the psychiatric literature, there is evidence to suggest that these effects may be important therapeutically. In an early study of Ketamine Psychedelic Therapy in patients with alcohol use disorders, negative experiences during the ketamine session (i.e., experiences associated with fear, anxiety, horror, and other negative emotions) were positively correlated with the length of remission (13). In a subsequent study where participants with heroin use disorders were randomised to receive either a “psychedelic” dose of ketamine [2.0 mg/kg Intramuscular (IM)] or a “sub-psychedelic” dose (0.2 mg/kg IM), the rate of abstinence over 2 years was higher in the former group than the latter (14) indicating a therapeutic benefit of ketamine-induced psychedelic experiences. Additionally, in a recent report antidepressant response was correlated with dimensions of altered states of consciousness such as feelings of unity, spirituality and insight (15). Moreover, ketamine's therapeutic effects on motivation to quit cocaine and cocaine use were mediated by ketamine's mystical type effects—but not dissociative effects (16, 17). Whilst some studies (18, 19) have described the experiences during treatment, there have been no qualitative studies of the experiences of participants during ketamine treatment for alcohol use disorders, and as far as we are aware of only a handful of studies that have looked at ketamine experiences qualitatively in any treatment setting (10, 11).

The participants in the present study were a subsample of participants from a recently completed randomised controlled trial, Ketamine for the Reduction of Alcoholic Relapse (KARE), which investigated ketamine as a treatment for alcohol use disorders (8). In this trial, three infusions of ketamine, compared to matched placebo, were found to be effective at prolonging abstinence from alcohol in recently detoxified patients with alcohol dependence, with the greatest benefit at 6 months observed in those who had also received psychological therapy. Whilst quantitative data are useful for establishing efficacy of novel treatments, qualitative data about patient experiences may provide insight into potential mechanisms of such treatments, particularly in newly developing fields such as psychedelic treatment. Additionally, qualitative data can provide important additional information alongside psychometric measures from clinical trials such as motivations and experiences of ketamine treatment which can inform the design of future trials. In the current study, we investigated the retrospective subjective experiences of participants who received at least one ketamine infusion and psychotherapy/psychoeducation as part of this clinical trial. Through semi-structured interviews we aimed to explore participants' acute psychological experiences under the ketamine infusions and perceived long-term effects of ketamine treatment and the overall trial.

## Materials and Methods

### Design

The current study involved semi-structured interviews which lasted up to an hour and a half each and were conducted by two members of the team (O.M.M., J.K.). Before commencing the interview, participants were invited to guess which condition they had been allocated to, as they had been blinded up to this point. The interviews comprised of three main sections: questions based on acute and subacute ketamine experiences, experiences of rumination (reported elsewhere), and current drinking levels (see Appendix for the interview schedule). Participants were invited to elaborate on their experiences using prompts and were invited to share any other experiences they thought were particularly important.

### Participants

Twelve participants (nine males and three females) who had previously taken part in a Phase-II double blinded randomised controlled multisite trial in London and South West England were recruited in this follow-up study. The main inclusion criteria for the trial were being 18-60 years old, meeting the Diagnostic and Statistical Manual of Mental Disorders (DSM)-V criteria for severe alcohol use disorder or DSM-IV criteria for severe alcohol dependence in the past 12 months, currently abstinent from alcohol, and a negative urine drug screen. Individuals on other relapse prevention medication or antidepressants, those with uncontrolled hypertension, and those with history of psychosis or first-degree family history of psychosis were excluded [Full criteria are reported in McAndrew et al. (8)].

### Setting

The aim of the KARE trial was to assess the efficacy of ketamine infusions combined with psychotherapy or alcohol education on reducing relapse rates in recently detoxified alcohol dependent individuals. In this trial, participants were given ketamine (0.8 mg/kg) or saline placebo infusions weekly for 3 weeks alongside seven sessions of either psychological therapy or alcohol education. The therapy or education sessions were always timed immediately before the infusion and ~24 h after the infusion. Prior to each infusion participants were read a script to prepare them for the ketamine experience, which included a suggestion to consider an intention for the session if they wanted to. The infusion was administered by a blinded anaesthetist through a cannula in the arm. During the infusion participants reclined on a bed in a single room with dimmed lights, listening to soothing music on headphones. Full instructions provided to participants prior to their infusions can be found in the Supplementary Materials. The infusion lasted 40 min and a psychologist and a nurse were present throughout the infusion. Participants rated potential side effects before, during and after the infusion on a standard scale developed for ketamine. Participants were followed up at 3 and 6 months after the end of treatment. Further details of the KARE trial are published elsewhere (8). Out of 48 participants who had been allocated to the ketamine group, only 25 were contactable. This is because the consent forms outlining whether they had consented to being contacted about future research were not accessible for all participants at the time due to one of the clinical trial units being closed during the COVID-19 Pandemic. These individuals were invited to take part in this qualitative research and offered £30 reimbursement in online vouchers for their participation. Ethical approval for this study was obtained from the Institutional Ethics Committee at the University of Exeter (Reference no: eCLESPsy001453 v7.1).

### Procedure

Participants were contacted with an information sheet and consent form for the study. Each participant was given a participant ID and the data collected as part of the study was stored separately from the consent forms. After giving informed consent, participants who received at least one ketamine infusion were invited to an online interview to gather data on their acute experiences during the ketamine infusions. All interviews were conducted online over Zoom, each interview was audio recorded in its entirety and was transcribed verbatim by Zoom. The recordings were checked by researchers for transcription accuracy. Participants' personal details were removed from the transcripts to preserve anonymity and the audio recordings were deleted once the interviews were transcribed and checked.

### Data Analysis

The qualitative data were analysed with Reflexive Thematic Analysis (TA). Reflexive Thematic Analysis as defined by Braun and Clarke (20), seeks to identify patterns of meaning across a dataset. It involves using qualitative methods of data collection and analysis, within a qualitative paradigm (20, 21). It is theoretically flexible and can be used in different frameworks to answer different questions (20). We adopted a realist/essentialist approach, whereby we assumed a straightforward relationship between language and meaning. We initially sought to identify participants' experiences of ketamine at different stages of the trial; pre-trial, acute experiences, and subacute experiences. Our analysis was partly theoretical in that it was conducted within the lens of current research on ketamine and its effects. However, we endeavoured to stay as close as possible to the participants' experiences and in doing so uncovered some effects of ketamine previously not reported in other quantitative studies.

We followed the six stages of analysis identified and developed by Braun and Clarke (20, 22). Two researchers reviewed the transcripts numerous times to familiarise themselves with the data. Initial codes were developed following a line by line reading of the transcripts. After coding transcripts independently, the researchers compared and discussed any discrepancies in coding labels and conceptualisations of the codes. A consensus was reached between the two researchers for each code, disagreements between the two researchers were resolved by discussion with a third researcher. Codes were then analysed further to identify overarching themes underpinning the data and develop a thematic model. Identifying themes was an iterative and collaborative process involving the whole research team.

All researchers involved in the process considered how their knowledge and experience might be impacting on the research at all stages, as they wanted to stay close to the participants experiences. Memos and research notes were kept to record this process of reflection and this was a regular point of discussion in research meetings. Literature on ketamine was partly drawn upon to structure the analysis as we initially explore experiences before trial participation, during acute and subacute effects of ketamine, and the long term effects of ketamine and the trial.

## Results

### Participant Characteristics

The demographic and clinical characteristics of the sample who took part in the interviews are reported in Table 1. Ten participants reported their age, which at the time of interview ranged from 22 to 59 (*M* = 46.5, *SD* = 11.1). One participant identified as Scottish Iranian, and the rest were White. The time from last infusion to interview ranged from 11 months to 3 years and 4 months. All participants except one were in employment at the time of interviewing. Demographic details of the full sample and results of psychometric measurements will be available in the publication of the main trial data. Four of the twelve interviewed participants had received the psychotherapy as part of the trial and the rest took part in alcohol education sessions. All participants interviewed here correctly guessed their allocation in the ketamine group.

**Table 1:** Demographic and clinical characteristics of participants.

**ID**	**Age**	**Gender**	**Ethnicity**	**Site**	**DSM-V criteria at baseline**	**Total number of infusions**	**Total number of psychotherapy/ education sessions**	**Psychotherapy or alcohol education allocation**	**Current drinking days per month**	**Current craving levels (ACQ-SF-R)**	**Abstinence following the trial**	**Reasons for breaking abstinence**	**History of anxiety**	**History of depression**	**Time from last infusion to interview**
P02	59	Male	White	Exeter	4	3	7	Psychotherapy	20-25	2.74	39 days	Social event	No	No	2 years and 8 months
P03	47	Female	White	Exeter	9	3	7	Psychotherapy	0 (Abstaining)	3.45	Fully abstinent	N/A	Yes	Yes	1 year and 8 months
P04	53	Male	White	Exeter	7	3	7	Alcohol education	8-10	2.58	40 days	Desire to drink	No	Yes	1 year and 1 month
P05	53	Male	White	Exeter	10	3	7	Psychotherapy	0 (Abstaining)	2.08	Fully abstinent	N/A	No	No	11 months
P06	56	Female	White	London	5	3	7	Alcohol education	20	2.45	2 months	Celebration	Yes	Yes	1 year and 8 months
P07		Male	White	London	7	3	7	Alcohol education	0 (Abstaining)	3.55	6 months	Stress and low mood	Yes	Yes	1 year and 1 month
P08	42	Male	Scottish Iranian	London	9	3	7	Alcohol education	8	4.57	1-2 months	Social event	No	Yes	2 years and 3 months
P09	35	Male	White	London	9	3	7	Psychotherapy	2	3.11	1 month	Social event	Yes	Yes	1 year and 2 months
P10		Male	White	Exeter	9	3	7	Alcohol education	3-4 days (Abstinent except for a recent binge)	2.97	14 months	Stress and low mood	Yes	Yes	1 year and 1 month
P11	50	Male	White	Exeter	10	3	7	Alcohol education	14	2.58	12 days	Social event	No	No	2 years and 6 months
P12	22	Male	White	Exeter	7	3	7	Alcohol education	15-20	3.5	2 months	Social event	No	No	2 years and 4 months
P13	48	Female	White	Exeter	7	1	2	Psychotherapy	28	1.34	Not known	N/A	Missing	Missing	3 years and 4 months

*DSM-V, Diagnostic and Statistical Manual of Mental Disorders 5th version; ACQ-SF-R, Alcohol Craving Questionnaire Short Form Revised. The participant allocated ID number 01 was subsequently not available for interview*.

At the time of enrolment in the KARE trial, participants had met a mean of 7.7 (*SD* = 1.91) DSM-V criteria. Current DSM V criteria were not measured at the time of interview, however we recorded current drinking days per month and current craving levels (see Table 1). Craving scores on the Alcohol Craving Questionnaire Short Form—Revised ranged from 1.34 to 4.57 (*M* = 2.91, *SD* = 0.82). Out of 12 participants interviewed here, three were completely abstinent at the time of the interview and one was abstinent except a recent binging episode. Following the trial, length of abstinence ranged from nearly 2 weeks to 14 months for those who were no longer abstinent, two participants had completely remained abstinent since their participation in the trial. Reasons for breaking abstinence following the trial included mainly social events, stress and low mood, desire to drink and celebrations. The rates of abstinence from alcohol use in the total sample of the original KARE trial (*N* = 96) will be reported in the publication of the main trial. The characteristics of the sample interviewed closely resembled those of the total sample (*N* = 96). Though a higher proportion of participants interviewed here had received alcohol education compared to psychotherapy, which was allocated on a 1:1 ratio in the trial.

### Qualitative Results

Six key themes were identified following our thematic analysis of all interviews, which explored participants' experiences at all stages of the trial (prior to the trial, acute experiences, subacute experiences, and post-trial effects). These themes, which are explored in detail below, include (See Table 2):

**Table 2:** Themes, codes, example quotations.

**1. Multifaceted motivations for seeking ketamine in a clinical trial**	
Concern over alcohol use and health	“*I was aware I was drinking too much, and it looked like a very interesting way of beginning to address it*.” (P13)
Hitting rock bottom	*“.for me, it was kind of it felt like kind of like the last chance saloon really to kind of do something.”* (P09)
Altruism	“*… I thought what if the ketamine trial proves successful and you know it just saves one life, someone who might die because of alcohol dependency issues, and then it is worth my continuing*.” (P04)
Legitimacy of the trial and curiosity	“*I used to have a personal trainer and I noticed he posted on Facebook actually the KARE trial was happening. And that's why I saw about it and I just got intrigued by it*.” (P11)
**2. Set and Setting as influential in determining acute ketamine experiences**	
Set	“*. I've done that before with ketamine but in a gang of friends, which is kind of what you do with friends and it's all a bit sloppy and daft after a big techno night and it's not something I've done much in the last five years before the trial anyway. But you know, I do know that world and they're lovely*” (P06)
Setting	“*I had no idea what would happen. but then after that, the second and third I sort of went into the sessions with more intent to explore and to get answers from certain things. it was nice to just do that and know that you're in a safe environment and you can come out the other side. It filled you with confidence and you know there's no paranoia*.” (P10)
**3. Inherent contradictions of acute experience**	
Positive effects	
A. Calmness and relaxation	“*It was peaceful. It was calming and I just sat back and. you know, I didn't lose sense of who I was. I always knew who I was, and I had confidence that this would be temporary*.” (P04)
B. Reinforcing effects	“*I wanted this to carry on every week for the rest of my life. I wanted this experience in this, you know, sort of clinical place, I would still be coming now you know what I mean?*” (P05)
Negative experiences	
A. Fear or panic	“*I knew where I was, but I just, it wasn't a pleasant experience like the first one and then the end part, the second one. It was, it was scary. And to the point of when you're on it, you barely breathe, you've quite shallow breathing*.” (P03)
B. Paranoid ideation	“*So it was a sort of mixture of extreme comfort for want of a better word with a sort of paranoia where one's brain is saying, if you guys in the room will leave the room, I'm stuck here for the rest of my life, sort of thing*.” (P02)
C. Paralysis	“*It's almost like a state of paralysis, where you do receive visual and sound information. But I don't know how much sense I was making to the people in the room around me. It feels like when you try and run in a dream, but your legs are move fast enough. In fact, exactly like that*.” (P08)
D. Nausea and vomiting	“*I was I didn't feel particularly brilliant, to be honest. And then I started moving and just was aware I was going to be sick. And then I was quite violently sick. So that was my experience*.” (P13)
Otherworldly experiences	“*Well, that infusion took me somewhere that was out outworldly, was out of this world. It was not—it was not within this world*.” (P05)
Dissociation, detachment, and floating	“*… as far as I was concerned that was it. I was done. My human body wasn't—I didn't have a human body. I was something… And even though I knew that I was kind of tethered, I didn't know how I was going to get back to my body*.” (P05)
Ego dissolution	“*The experience was genuinely remarkable in terms of the both the visual effects and…. the sort of removal of ego that accompanied that. I felt that I was one with the whole universe and it sounds hippy dippy but that's how I felt. I think I related to this sort of ego changing, the size of the ego in me as well as a sort of physical sensation*.” (P07)
Changes in experiences over time	“*So, the first one was very trippy and like going into another dimension. And then the second one, because I wanted to go into that dimension, I think I was trying to go there and then I got a bit sort of like, oh, I can hear voices. This is putting me off. And then the third one. I wasn't well at all after that. As I said I couldn't come around from it*.” (P03)
**4. Rapidly fluctuating and changing experiences**	
Perceptual distortions	
A Visual distortions	“*I remember looking at the light above my head in the window and sort of blinking and as I blinked the of the colours changed. Even though. the picture in my mind, the light in the window was the same*.” (P07)
B Auditory distortions	“*.there were pieces that I was familiar with, but they were just, they were coming—It's like different aspects that made up the piece were coming through a different speeds or different pitches. They were just sounding like unique. … I could still hear them as piano pieces but they weren't the pieces that I knew*.” (P04)
C Hallucinations or visions	“*I saw.this very strong vision of the synapse travelling across the neural pathway, then me typing into my phone, him getting the message him talking to someone else, and like this chain of communication, essentially that got bigger and bigger and bigger and bigger and bigger, to the point where it zoomed right out and you're seeing the earth hanging there in space along with say 20, 25, 30 other inhabited planets all there in the cosmos*.” (P09)
**5. Meaningful, spiritual, and mystical experiences**	
Ego dissolution	“*It was a sense of completeness sense of, I suppose in a way finality, a source of finish. But also, a sense of enormous growth and a feeling of oneness with other entities, other living beings in particular, but also the world and universe as a whole*.” (P.07)
Epiphanies and Enlightenment	“*I think the first two like, they sort of left me… like they answered a lot of questions. You know, thinking about my children and I have a stepdaughter and… It was things about that. And about, you know what I should be doing and how I should be, you know, I don't know it seemed to be like all the things that are really heavy on my mind and that I stress about whatever it was sort of going through those things.just realising how little importance some things had or were*.” (P10)
Transcendence of time	“*.while we're on the subject of time. I mean, that just goes bananas and I love it. It's almost like. this afternoon, this evening, the way we think of that—That just blows apart and doesn't exist anymore*.” (P06)
Hallucinations or visions	“*I was lying there, and there was this, there was this like a cacophony coming from the hallway, everyone was going like, like he's coming he's coming! And I was like what's going on, you know, like thinking this shouldn't be happening in the hospital, I thought this is a peaceful place and everyone's like rushing out into the hallway to see what's happening. And it's this cartoon very simply drawn constructed of neon light rendering of God, essentially is walking down the corridor.and he came up to me and he was like.you can ask me two questions*.” (P09)
**6. The ketamine infusions and the trial as transformational**	
Perspective on life	“*It helped family wise, relationship wise in every, every single avenue of my life. It's changed it.doing the ketamine and seeing this other dimension enforced my belief of another life and I now live every single day to the max. When I go for a walk, I'm very observant of my world around me. I take pleasures in life rather than pleasures of.drink. So.it's still with me and I hope it'll stay with me for forever*.” (P03)
Relationship with alcohol	“*I think before the trial all my life was sort of focused around alcohol. I was either drinking it at home or selling it to students or working in an event where there was alcohol, the alcohol was a focus of it. So it was sort of everything and then afterwards, it just sort of stopped. I enjoy a drink every now and then, but under much safer ways really.So it just made me realise I don't need to sort of drink to excess because there's nothing else to do. I can just do other things.and that alcohol isn't everything*.” (P12)
Positive experiences of psychotherapy and alcohol education	“*You definitely need that support system. It feels like that you get with the therapy and the fact that you can take that home with you as a crutch. So yeah, it was, it was very good, obviously painful in some parts, but you know I was at my most. desperate. I wouldn't be here now, if it wasn't for it. I can definitely say that*.” (P03)
Interaction of ketamine with psychotherapy or education	
A. Ketamine interacting with psychotherapy or education	“*If I had just gone to education sessions and gone: ‘oh well I work around alcohol all day, I am pretty sure I know all the stuff I need to know about it,' I probably wouldn't have listened so deeply. But the ketamine sort of made me more willing to engage with it*” (P12)
B. No impact of ketamine on psychotherapy or education	“*I don't think there was any correlation there because the effects had fully worn off before we would go into that kind of conversation*.” (P08)
Ketamine and talking sessions as mutually supportive	“*I was kind of more open to understanding those mental and physiological impacts of alcohol because of the mental effects I've had from the ketamine. Um, so I think there was there was a sort of mutually supportive relationship between both elements to the therapy*.” (P07)
Non-specific trial effects	“*It's more the sort of holding hands that one needs, you need somebody who you've sort of divulged all your innermost secrets to. Having done that, it then sort of makes you think twice about buying that bottle of wine. It's that sort of attention that you get that I think is valuable in this sort of thing*.” (P02)

1. Multifaceted motivations for seeking ketamine in a clinical trial.
2. Set and Setting as influential in determining acute ketamine experiences.
3. The inherent contradictions of the acute ketamine experience.
4. Rapidly fluctuating and changing ketamine experiences.
5. Meaningful, spiritual and mystical experiences.
6. The ketamine infusions and the trial as potentially transformative.

#### Multifaceted Motivations for Seeking Ketamine in a Clinical Trial

Participants' motivations to take part in the trial were often multifaceted, with participants citing more than one reason for their participation. The key motivations for participation identified included: concern over their own alcohol use, hitting rock bottom, altruism, legitimacy of the trial, and curiosity. Motivations were both internally focused (e.g., participant's recognising their relationship with alcohol meant they needed to take action to benefit their health) and externally influenced (the legitimacy of the trial at a University and hospital encouraged participation).

##### Concern Over Alcohol Use and Health

Participants were motivated to take part in the trial when they assessed their own health as requiring action: “*I was looking for ways to just become a lot healthier. And to become just absolutely teetotal*” (P08). For the majority of participants, concerns over alcohol use were a key motivator for taking part.

##### Hitting Rock Bottom

Some participants felt that their alcohol use was beginning to “*spiral out of control*” (P12) and reported feelings of hopelessness and suicidal thoughts related to their level of drinking, which led them to engage with the trial. In this way the trial was described as a “*last chance*”: “*I was at my wit's end, at my lowest point. I had no escape, no way out of alcohol to the point of I knew if I carried on, it would kill me*” (P03).

##### Altruism

For some participants it was also the potential to help others through taking part in this clinical research trial that provided extra motivation for them to sign up: “*My thought behind this process was that if I did these 6 months and it helped others. That's something that I, I, you know, it's a little bit of extra incentive for me”* (P05).

##### Legitimacy of the Trial and Curiosity

The legitimacy of the trial afforded by it being conducted at a well-known University and a hospital, seemed to provide reassurance for participants and sparked their curiosity to participate:

“*I sort of tend to be a little bit cautious with these sorts of things, but because it was sort of university and hospital based, I thought well you know, why not give it a go. The risks seem very small*” (P02).

Indeed, many participants reported curiosity related to the trial advertisement, noting in particular, interest in exploring ketamine therapy as part of a clinical trial setting.

#### Set and Setting as Influential in Determining Acute Ketamine Experiences

##### Set

Participants' expectations of the infusions, elicited from past experiences of changes in consciousness with and without drugs, had an impact on their acute experiences of ketamine. Similarly, the absence of prior drug experience exerted an influence on participants' acute experiences. For some participants who had not used drugs recreationally in the past, the ketamine experience was particularly novel. For one participant, speaking to a friend who had used hallucinogenic substances prior to their second infusion helped them to feel more “prepared” for the experience:

“*And he advised me just to relax, he said, just chill out. Just have the confidence that you're gonna you're going to get out of this. And the whole situation will be more, will be better for you. And basically, yeah that's. I was prepared for it*” (P04).

Participants' prior mindset and spiritual beliefs were also reported to affect their acute ketamine experience: “*And for me, the idea that science and a form of spiritual practise or awareness are two wings of the same bird. So, I think that probably informed how I experienced this*” (P09).

Participants' experiences during the trial and expectations following the first infusion also appeared to affect their experiences of the following two infusions. For instance, one participant recounted that concerns by the trial staff about their mood following the first infusion led to a discussion on whether they can continue with the infusions, and although they were able to continue, this experience impacted their next infusions:

“*The process of getting back on the trial and having to fight for it meant that I was carrying some sort of negative feelings. And that's slightly obscured those positive aspects that I had before [during the first infusion]* … *I was nervous and that affected the experienced I had*.” (P07).

##### Setting

The setting in which the trial took place was described as “professional,” “controlled,” “carefully regulated,” and “clinical.” As participants reported that the legitimacy of the trial was one of the reasons that motivated their participation in the trial, related to this they reported feeling safe and being comforted by the professionalism of the trial staff as well as the clinical setting in which the trial took place: “*I thought it was amazing how clinical it was and how organised it was and how safe I felt with it. That was brilliant*” (P11).

This professional and regulated setting of the trial was reported to play an important role in determining participants' acute ketamine experiences:

“*I just thought, right* ‘*How extreme is this experience going to be? Is it going to be a ride from hell or something like that?*” *And it absolutely wasn't and that's down to the laboratory type conditions, the controlled environment*” (P08).

#### The Inherent Contradictions of the Acute Ketamine Experience

The acute ketamine experiences of our participants were characterised by inherent contradictions. Participants described experiences that were both highly positive and negative and could be conceptualised as a “rollercoaster ride.” It is also worth noting the negative experiences were largely transient and that despite these negative experiences, most participants described their acute ketamine experience as overall pleasant: “*I just found I left every session, kind of, you know, feeling happy. Feeling, you know… feeling lighter… Feeling like clearer thoughts*” (P10).

This “rollercoaster” was experienced differently by each participant and whereas for some, experiences were largely positive, for others the rollercoaster led to a real divergence of positive and negative effects, all occurring in the context of differing/vivid psychedelic experiences, as we will discuss further below.

##### Changes in Experiences Over Time

Moreover, exploring participants' experiences across the three infusions highlighted further contradictions—for some participants the infusion experiences were qualitatively similar, others described each infusion having a unique effect:

“*The first one was in the imagination., with what I know as a human being, got my mind my will being put on it…. And the second one was just freedom and just wow…. The third one was a little bit too short, and as I came out I was panicky. I was just like ‘that's it, we're done*”' (P05).

Some participants perceived the experiences as less vivid/potent as the trial progressed due to the reliability of the effects: “*I think having, having had it a couple of times, I was kind of probably more used to what I was expecting. I suppose it was less of a wow, this is amazing, because I've done it before*” (P07).

This reduction in the strength of effects across the infusions was reported to lead to dissatisfaction due to expectations from previous infusions and a desire to replicate such experiences: “*My body was still starting to disappear, but not as quick so. It's almost like your body gets used to it*” (P03).

##### Positive Experiences

**Figure 1:**
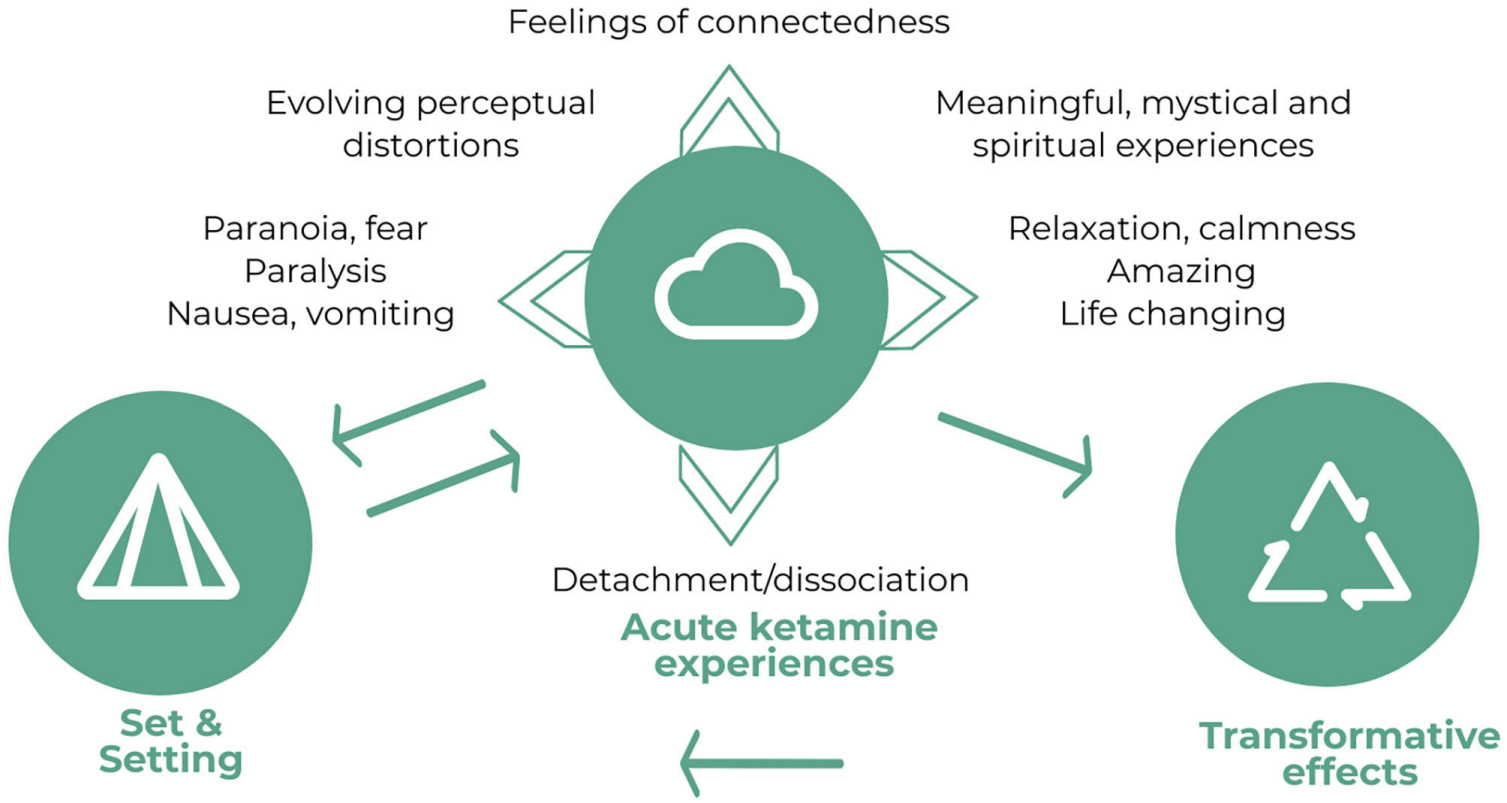
Diagram depicting the relationship between set and setting, the acute ketamine experiences (inherent contradictions of the experience, rapidly fluctuating and changing experiences, and meaningful, spiritual and mystical experiences) and transformative effects of the infusions and the trial. Whilst set and setting are influential in determining the acute ketamine experiences, experiences from the first two infusions and the transformative effects also contribute to the set of the subsequent infusions as expectations. As discussed in theme 4, the acute ketamine experiences, particularly feelings of connectedness and dissociation also appeared to be related to the transformative effects.

As seen in Figure 1, positive effects identified during the infusions included feelings of calmness and relaxation. Particular experiences were feeling relieved, unhooked, mellowed, carefree, chilled, and peaceful:

“*It was, um, it was a sort of sense of complete relaxation, a sense of sort of vanishing, you can, it feels like one was being sort of enveloped in a duvet type, you know, that sort of feeling, if you like*” (P02).

Another positive aspect of the experience was the reinforcing effects of ketamine. Participants described the experiences associated with the ketamine infusions using terms such as “beautiful,” “fantastic,” “brilliant,” and “amazing.” Several participants reported the experience as “life changing” or “one of the best experiences of my life.”

Due to the positive effects of ketamine infusions, feelings of loss and sadness were described in relation to the cessation of the ketamine infusion and the trial. Participants reported wanting the experience to continue, or that they would benefit from a larger number of sessions (e.g., six rather than three); this was related to the changes in experiences across infusions which meant that each infusion led to a deeper understanding:

“*Because there's the three sessions, really like I wanted to do six thinking back on it because I felt like the third one, I just really cracked into something and you know to go a little bit further, I think would have been really good*” (P09).

##### Negative Experiences

On the other hand, the transient negative effects included fear or panic, mild paranoid ideation, paralysis, and nausea and vomiting. Nearly all participants experienced some form of transient psychological or physical distress during the acute ketamine infusion. Participants commonly described fear of permanently losing their sense of self and body. This fear was attributable to a sense that the trial would never end: “*It was the fear factor of I couldn't get out of it. I couldn't come off it*” (P03).

Despite reports of feeling safe and comforted by the professionalism of the trial staff, several participants started to question the veracity of the trial and the motivations of the trial staff whilst under the acute effects of ketamine: “*And I thought, hang on, what have I let myself in for you know? What is this thing? What the hell is going on here?*” (P08).

These thoughts and feelings of paranoia were described to be “strong” and “overwhelming.” Additionally, these thoughts were linked with beliefs that they would be stuck in this situation for the rest of their lives:

“*I can remember thinking, feeling very paranoid that the whole trial was kind of a fake and it was like trying to get me into this situation. And that I was actually going to be in a situation for the rest of my life*” (P04).

The transient physically distressing experiences took the form of paralysis and nausea and vomiting. A few participants described feeling nauseous and sick during the infusions. A number of participants reported varying degrees of paralysis, whereby they were not able to move parts of their body due to feelings of detachment:

“*And it's almost like a state of paralysis, where you do receive visual and sound information. But I don't know how much sense I was making to the people in the room around me. It feels like when you try and run in a dream, but your legs aren't moving fast enough. In fact, exactly like that*” (P08).

Another set of contradictory experiences involved on one hand experiences of dissociation, detachment, floating, otherworldliness and on the other hand ego dissolution characterised by feelings of connectedness with other beings, decreased self-importance, a sense of the small self-fitting in with the vastness of the universe.

##### Dissociation, Detachment, or Floating

Nearly all participants reported feelings of dissociation or detachment, either from their environment, their physical body, or their sense of self: “*I kind of lost a sense of who I was. I couldn't—there didn't seem to be no connection with normality and what, how I usually felt*” (P04).

Other experiences of dissociation involved participants feeling detached from their physical bodies or feeling as though their bodies had disappeared: “…* My whole feeling of my body disappeared…. It was like being an amoeba and just floating in space. I had nobody, I had no- I was a soul*” (P03). For some people this experience of was accompanied by feeling of paralysis, which was reported as disturbing, as intense feelings of detachment made them feel like they had no physical connection to their bodies: “*One further thing I remember is I couldn't actually move any part of my body because of the detachment*.” (P04). In an extreme case of detachment from their physical body, one participant reported what they described as a near death experience: “*I was this entity of pure whiteness, and I was above myself. As far as I was concerned, that was it. I could be dead. I could have been that was it. I could have stayed white forever*.” (P05).

##### Otherwordly Experiences

Participants also reported experiences which can be described as dissociation from the world; ketamine infusions were commonly described as “out of this world,” “otherworldly” or opening one up to a “new realm”: “*That infusion took me somewhere that was outworldly, was out of this world. It was not within this world*” (P05).

##### Ego Dissolution

In direct contrast to these experiences of dissociation or detachment from one's sense of self, physical body or the world around them, experiences of ego dissolution were reported; these were characterised by a diminishing sense of self as distinct from other beings and feelings of unity with the rest of the universe. Some of these experiences were primarily characterised by decreased absorption by one's own issues and concerns and a decrease in sense of self-importance:

“*I felt a sense of all the things that I decide or wanted or had or didn't have, was kind of understandable in a sort of childish way but actually not what was important. I didn't have any acquisition, or requirements or needs. I was over that. It was a sense of completeness, a sense of, I suppose finality, a source of finish*” (P07).

Whilst for others, this experience of decreased self-importance and decreased self-absorption was linked to a feeling of connectedness with other beings, a sense of the “small self” and/or fitting in within the vastness of the universe:

“*It was almost like the universe was surrounding me and I was just the most tiny you know, small, small and fundamental particle, you know. From this feeling of like this huge vastness and me being absolutely nothing or very, very, extremely small. And because of that tininess of me, it was almost like all the things that made me no longer existed*” (P04).

#### Rapidly Fluctuating and Changing Experiences

##### Perceptual Distortions

All participants reported perceptual distortions, which were variable in nature and changed throughout the experience. These took the form of visual and auditory distortions and hallucinations or visions. Visual distortions were most commonly reported, which included dimensional and spatial distortions, whereby objects and people felt smaller or larger, closer, or further away, or had a different shape or texture: “*I could feel different textures of surfaces. I could see movement of these surfaces like snakeskin scales or lava. And different shades of colours*” (P07). For some, this involved feeling themselves as smaller or larger in comparison to the room. “*I got a sort of spatial sense of being enormous or being tiny*.” (P07). Aspects of texture and colour were also altered for these participants. Most participants reported changes in colour perception, which was described as “beautiful” and an “explosion of colour.”

Others reported auditory distortions, such as voices sounding further away or closer and musical pieces being unrecognisable “*It's like different aspects that made up the piece were coming through at different speeds or different pitches. … I could still hear them as piano pieces but they weren't the pieces that I knew*” (P04); or changing depending on their concurrent psychoactive experience: “*So, when the music tempo picked up, it's sort of moved into a different place within the journey that you were on essentially*” (P11).

These experiences of visual and auditory distortions were reported to change and evolve throughout the infusion, this is key to the rollercoaster of experiences whereby perceptions and reality are rapidly changing:

“*Sometimes it would become very linear, everything will be at right angles and lines and squares. And other times it would be just like a blur it would just be colours. And it would constantly change and evolve, what I was seeing would evolve and what I was hearing would evolve as well*” (P04).

On the other hand, for some the sensory changes were more extensive than simple distortions, crossing over into what can be described as hallucinations or visions. These were very subjective, ranging from research staff transforming into Alice in Wonderland characters, images of being surrounded by hundreds of people, images of being surrounded by pink blancmange and abstract hallucinations of a biological nature:

“*So, everybody turned into Alice in the Wonderland for me. … And then the other one I can't remember her name, she is wonderful, but she was the Cheshire cat. So, when she walked in, she had this massive grin*” (P05).

#### Meaningful, Spiritual, and Mystical Experiences

##### Ego Dissolution, Epiphanies, and Enlightenment

For several participants, these experiences of ego dissolution during the ketamine infusions were connected to gaining deep and meaningful insights to important aspects of their life (see also theme 4 below). For some, this took the form of gaining insights into a sense of what was important in life:

“*It was about material things, not wanting those, those aren't really important. And neither is the sense of doubt and self-criticism, you know, it's almost like life's too short. In a way, I felt like life was over. I can let these things go; you know that they are not really that important. Almost like simplistically, don't sweat the small stuff* ” (P07).

For one participant the epiphanies involved reflections on life and death:

“…*Life and death is not a black and white thing. It's not a switch on and switch off when you die. They are a passage and I'm a part of it here and I'm a part of it whenever that other thing happens*” (P06).

Another participant experienced an epiphany about many aspects of their life including an understanding about the need to integrate traumatic experiences within oneself; as we explore in the next theme, for this participant, these new understandings led to significant transformations in their life:

“*So, if trauma was like a ball so like you've got something about the size of a tennis ball that has a trauma experience. It's attached to your body. You can move it around in the body, but it's still attached to you. It's unpicking that fabric and weaving the fabric into your being. So, it doesn't cease to exist, but its power is gone*” (P09).

##### Transcendence of Time

Some participants reported experiences where a sense of time as we know it was eliminated. For some, distortions of time produced the fear of the experience lasting for eternity. For others, distortions of time had a more profound effect and put into perspective the normal ordering of events in society: “*Now, this afternoon, this evening, the way we think of that—That just blows apart and doesn't exist anymore*” (P06).

##### Hallucinations or Visions

For some, hallucinations and visions were related to religious, spiritual, and mystical experiences:

“…*.we are all connected and there is this connection between all beings, people and things to again bring us out of this kind of prison of addiction. The transpersonal effects of the drug bring us out of ourselves and put the problems into perspective*” (P09).

For one participant this took the form of encountering and talking to God. He described these experiences as “holy” and these visions were closely related to epiphanies:

“*And so essentially, it was like this higher power, this representation of the higher power [referring to God] that had appeared at the start was showing that I had permission to be happy and do I what want, as long as I didn't misuse my resources*” (P09).

It is important to note that every participant reported these acute ketamine effects as being transient in nature, with the only psychoactive effects in the following few hours being mild, such as “strange” feelings, slight feelings of confusion, or mild visual effects whilst going to sleep. Many participants reported that ketamine infusions had positive effects on their mood in the days following the infusions. These included reports of feeling “chilled out,” at peace, reflective, happy, energetic, or just generally being in a better mood but for most these positive effects on mood were brief and returned to baseline within a week.

#### The Ketamine Infusions and the Trial as Potentially Transformational

##### Perspective on Life

The combination of the ketamine infusions and the trial as a whole were reported to be transformational in many aspects of participants' lives: “*In a non-cheesy way, it actually probably changed my life around and kept me alive*” (P12). The effects of the trial were described as helping “every single avenue” of one's life (P03), as well as a “real fundamental shift in gears” (P09).

It's important to note that not all participants reported these transformational effects of the trial, several participants expressed that the trial did not lead to a fundamental lasting change on their perspective of life. Though one of these participants described the trial as a “major a part of a process” which allowed them to move forward with regards to their mental health.

##### Relationship With Alcohol

Whilst only three participants were still completely abstinent at the time of interview, all participants who completed the treatment recounted that the trial had transformed their relationship with alcohol in a number of ways. For some, this consisted of a switch from uncontrolled to a more controlled drinking approach, whereby participants were able to remain abstinent for much longer periods and consume much less alcohol: “*I still drink, but I'm quite capable of having 2, 3, 4, 5, 6, 7 days where I'm just not bothered about it, which is not something that happened in the past*” (P04).

For others, the transformational effects involved reduced craving or urge to drink alcohol, which was accompanied by reduced pleasure from alcohol in some: “*I feel I have much less desire to drink now than I used to. And I think what it is, I actually, I think, enjoy it less now*” (P11).

In some cases, the trial and the infusions resulted in changes in drinking motives from drinking to cope with negative emotions or boredom, to drinking for social reasons instead: “*It's more just sociable drinking now, not:* ‘*Oh I've got nothing to do after I finish work, I might as well just go get a bottle of rum and drink that”'* (P12).

For participants who were abstinent from alcohol at the time of interview, the trial led to more fundamental changes in their relationship with alcohol, where alcohol no longer was the primary focus of their life which meant that they were able to prioritise other aspects of their life: “*Whilst I was drinking, drink was the most important thing and drink was the dangerous thing. It was you know, my love and my hate, drink was and now I've got swimming*” (P03). This also involved a switch from wanting to find a reason not to drink, to longer needing a reason not to drink: “*Right now I've got to have a reason. Show me the reason to drink. I don't have a reason, I don't need a reason not to drink anymore*” (P05).

For those who were abstinent from alcohol, some of these transformational effects were closely linked with the deeply meaningful, personally relevant, religious, spiritual and mystical experiences, and dissociative effects during the acute ketamine experiences. For one participant, overcoming the near death like experience of extreme dissociation from their physical body became a coping mechanism for dealing with other problems in life:

“*So moving onwards in life, every time I get something that's quite testing or you know a problem, I just say, ‘well, a couple of months ago, I was just white…. So how bad can this be?'*” …* At least I'm not white, you know, at least I've got a body, at least I'm alive kind of thing* (P05).

Similarly, for another participant, the experience of disembodiment during their first infusion was connected to their abstinence from alcohol: “…*After the first one, I knew I wasn't going to drink again*.” (P03). Indirectly, this strong experience of dissociation appeared to be related to the participant forgetting the taste of their drink of choice and thus desire to remain abstinent: “*I don't recall what pint tastes like, now a pint was my drink. And I couldn't imagine the taste, so instantly I was not wanting to drink*” (P03).

For another participant, it was the reduction in self-absorption and feelings of connection with the universe that seemed to affect their relationship with alcohol:

“…*The sense of oneness that I felt and the sense of moving away from focusing on the worries and the small stuff is helpful in terms of improving my relationship with alcohol. Because I think I used alcohol as a self-medication and as a blocking and avoiding mechanism. And I think feeling that those issues are less prevalent or at least less important means I feel less motivated to drink*” (P07).

A number of participants who reported experiences of ego dissolution or epiphanies during the ketamine infusions were not completely abstinent from alcohol at the time of interview. Whilst they all reported changes in their relationship with alcohol as explained above, it is not clear whether these were due to their experiences of ketamine infusions or the education/psychotherapy: “*My attitude towards alcohol has changed, but I couldn't say how much that was down to infusions or education or a combination of the two*” (P04).

##### Positive Experiences of Psychotherapy and Alcohol Education

Another potentially transformative aspect of the trial was the psychotherapy/psychoeducation sessions provided alongside ketamine infusions. These were described as “illuminating” and “engaging.” Participants reported that even if they were vaguely aware of the dangers of alcohol, through the psychoeducation sessions they gained an in depth understanding about the detrimental effects of alcohol on the body and the mind. In some cases, this knowledge appeared to be linked to the transformations in drinking behaviour/attitudes to alcohol: “…* Actually learning the dangers of over drinking to go:* ‘*you know what, I don't really want to be around it that much anymore”'* (P12).

Only four of the participants interviewed had received the psychotherapy as part of the trial, nonetheless these participants recounted the transformational effects of psychotherapy, including opening one up to different kinds of therapy, as a support system that kept them focused on their journey, and as having significant impact on their personality:

“…*My persona was a jacket that I could take off and hang up at the door and do the work. And then put that jacket back on. But the jacket would be a slightly different colour and then by the end of the process, it was a different jacket*” (P09).

##### Interaction of Ketamine With Psychotherapy or Education

One possible transformational effect of ketamine infusions was making participants more receptive, more willing to engage with and more open minded towards the psychotherapy/psychoeducation sessions:

“*If I had just gone to education sessions and gone:* ‘*oh well I work around alcohol all day, I am pretty sure I know all the stuff I need to know about it*,' *I probably wouldn't have listened so deeply. But the ketamine sort of made me more willing to engage with it*” (P12).

Though for others, there was no obvious connection between their ketamine experiences and openness to learning new information as part of the talking sessions.

It is important to discuss whether the transformational effects reported here are due to the ketamine infusions, the psychoeducation/psychotherapy sessions, or non-specific trial effects for instance the care and attention that the participants reported receiving from the trial staff.

##### Ketamine and Talking Sessions as Mutually Supportive

Several participants described the ketamine and talking interventions as a package, components of which mutually supported each other, and attributed the effects of the trial to both:

“*So, I think that as a package, I hit the golden button, didn't I? …. Not only did I get a life changing and mind-altering experience, but then the therapist did plug some new thoughts to me that made me think differently. I feel that it is really important that when you are split open, you know, in such an intense and life changing way that you are given new thoughts and you know that someone gives you something to refill that, so you do change stuff* ” (P05).

##### Nonspecific Trial Effects

Others mentioned that the benefits they experienced may be due to other factors associated with the trial and highlighted that the emotional connection with the therapist and desire to do well for them also played a role:

“*I don't know whether it was the chemicals, the therapy, the keeping of the notes, the loyalty, the wish to sort of be a good pupil if you like in relation to the people involved in the course…. And I'm not sure it's the therapy that one needs. It's more the sort of holding hands that one needs, you need somebody who you've sort of divulged all your innermost secrets to. Having done that, it then sort of makes you think twice about buying that bottle of wine*” (P02).

Participants responded positively to the attention and care they received from the trial staff and their genuine interest in helping them: “*I did feel throughout the KARE trial, that one of the really positive aspects of the whole thing was the care and interest in me that I felt from the staff that I met with and engaged with*” (P07).

## Discussion

“*. so, it was showing that essentially, we are all connected. And there's this connection between all beings, people, and things to again bring us out of this kind of prison of addiction, the transpersonal effects of the drug to bring us out of ourselves and put the problems into perspective*” (P09).

“*I have to remember this, because this is going to be one of the best experiences of my life*” (P04).

This study set out to explore the subjective experiences of individuals in a clinical trial of ketamine for the treatment of alcohol use disorder. Our use of an open-ended, semi-structured interview allowed us to identify themes which may not have been apparent from using a standardised questionnaire or clinical checklist, and in doing so we were able to uncover a number of previously unreported effects of ketamine. This work has yielded important insights into the motivations for and experiences of ketamine treatment which may be useful for tailoring future treatments and preparing participants for ketamine treatment (see Table 3).

**Table 3:** A list of recommendations for future trials of ketamine treatment based on current findings.

**1. Preparation**
•Include first person accounts of ketamine experience in the preparation for infusions
•Emphasise that the experience may involve paranoid thoughts and altered perception of time, though any affects are transient
•Include a debrief at the end of the ketamine infusions to discuss potential feelings of loss and sadness associated with the end of treatment
•Screen individuals for tendency towards paranoid beliefs
**2. Setting**
•Clinical and professional setting may be reassuring for ketamine naïve participants
•Trusting relationships with the trial staff appear to be crucial in providing a safe setting
**3. Measurements**
•Include questionnaires measuring a wide range of the psychoactive effects of ketamine (religious, mystical, spiritual, and dissociative) including Hood's Mysticism Scale, Psychotomimetic States Inventory, and 5-Dimensional Altered Consciousness Rating Scale
•Measure motivations and expectations from treatment using measures such as The Stages of Change Readiness and Treatment Eagerness and The Alcohol Abstinence Self-Efficacy Scale
•Development of a new measurement capturing the wide range of acute experiences reported under ketamine infusions: dissociative, religious, mystical and spiritual, and otherworldly experiences as well as perceptual distortions
**4. Dosing and Administration**
•Consider titrating doses up according to individual experiences
•Consider multiple sessions based on individualised need/experiences
•Investigate the optimal number of doses

Consistent with previous work with psychedelics (23, 24), but rarely considered in ketamine studies, set and setting were found to be important in the acute experience following ketamine administration. Most of the respondents reported being comforted by the professional and clinical setting in which the trial took place. The setting of this trial represents a contrast with the way the therapeutic environment has been manipulated in studies of classic psychedelic drugs to feel comfortable and incorporate natural features (24–26). Whilst it was previously thought that clinical or medical environments may induce anxiety or unpleasant experiences (2, 25, 27), this did not reflect the experiences of the participants interviewed here.

The mindset or “set” was also found to be important: those who had previous experience of non-ordinary states of consciousness had found these experiences to be useful preparations. These reports parallel research in the context of psychedelics, which showed that readiness to surrender to the experience and being supported by trusted individuals in a therapeutic setting protects against challenging psychological experiences (24). The experiences reported by participants here further underscore the importance of the context in which psychedelic drugs such as ketamine are administered. Whilst previous research investigated the effect of drug by environment interactions on the acute experiences of psychedelic drugs (28, 29), future research is needed to examine the role of set and setting in determining the treatment efficacy of psychedelic drugs (30).

Participants' reported that ketamine induced religious, spiritual, and mystical experiences. This may be relevant to the use of the drug in this group of participants with alcohol use disorder, as connection to a higher power is a key element in some approaches to achieving and maintaining sobriety from alcohol, for example the twelve-step programme. It is possible that ketamine or other psychedelics might once again have a place alongside these treatment approaches (31) for those who have struggling to engage with these aspects of these programmes. Whilst previous research on religious, spiritual, and mystical experiences (RSME) has dismissed such experiences produced by psychedelics as artificial compared with spontaneous RSMEs (32), others argued that the “fruits” (outcomes) of the experience are more important than its “roots” [cause/origins as William James cf. (33)] (33, 34). In fact, a previous study has reported that the RSMEs induced by psychedelic substances were perceived as more mystical than RSMEs produced through other means and had greater positive impact on one's sense of purpose, and greater increases in individuals' spirituality (33). The participant experiences reported here are in line with previous research demonstrating that psychedelic drugs such as ketamine can trigger profound RSMEs when administered in highly controlled and supportive clinical trial settings (25, 30, 35–38). The extent to which such experiences are related to ketamine's treatment effects for addiction remains to be further investigated.

A number of participants reported ego dissolution from ketamine, which comprised feelings of connectedness with the universe, and a sense of the “small-self” fitting in with the vastness of the universe; similar to experiences commonly observed with other classic psychedelic drugs such as psilocybin (1, 30, 38). Whilst feelings of dissociation and detachment that were reported by the majority of participants are consistent with previous reports (39, 40) in the current trial these feelings of detachment from one's sense of self or one's physical body were accompanied by seemingly paradoxical feelings of connectedness with the universe and of decreased self-importance and self-absorption.

Acute ketamine experiences reported by patients—feelings of connectedness, altered time perception, self-diminishment, perceived vastness, and physical sensations—all map on to the experience of “awe” which is an increasingly researched psychological construct (41). Awe has been proposed as a potential mechanism of action underlying the effects of classic psychedelic-assisted psychotherapy (42) and theoretically overlaps with mystical experiences (43), the small self and ego dissolution (44) and challenging experiences (45) under psychedelics. The ketamine induced profound sense of awe, wonder and connectedness may be of particular use in this group of patients, in helping individuals break the cycle of compulsive patterns of thinking that are a feature of alcohol use disorders.

Given the reported experiences here, the term “dissociative anaesthetic” appears insufficient to characterise the wide range of acute ketamine experiences captured in this study. In previous clinical trials of ketamine as a mental health treatment, the Clinician Administered Dissociative State Scale (CADSS) (46) was the most commonly used tool to assess the dissociative effects of ketamine (47). However, concerns were raised that the CADSS may be inadequate to measure such experiences as it has only been validated in populations with Post Traumatic Stress Disorder/ dissociative disorders (46) and it does not appear to fully capture the phenomenological experiences of ketamine administration (48). It is important to accurately measure the subjective experience following ketamine, as there is considerable research interest in whether the acute psychoactive effects of ketamine mediate its therapeutic effects. Emerging evidence suggests the mystical, dissociative and psychotomimetic effects characterising the ketamine's acute effects may contribute to its therapeutic benefits (16, 17, 49) parallel to findings in the psilocybin research which links mystical experiences to treatment outcomes (50–52). A recent review suggested this evidence to be inconclusive for ketamine but concluded that this may be due to the measurement tools used [for a review see (47)].

Whilst a number of terms have been used to describe ketamine and similar drugs, including hallucinogen (perceptual alterations), entheogen (producing mystical like experiences), psychotomimetic (modelling symptoms of psychosis), all seem to suffer from the issue of focusing on a single aspect of the experience at the expense of others (2). The term psychedelics may also not be preferable due to strong connotations with Western counterculture of the 1960s (2). Additionally, feelings of connection, epiphanies and the awe aspect of the experiences are not reflected in any of the proposed names. It may be that a new nomenclature for characterising the effects of psychedelic drugs is needed, whereby each drug might be described in terms of the extent to which the described categories of experiences are reported.

Nearly all participants experienced transient periods of psychological and physical distress during the ketamine infusions. Despite reports of trusting relationships with the trial staff, under the effects of ketamine a small of number participants questioned the veracity of the trial or the motivations of the trial team; these strong and overwhelming paranoid thoughts were linked with beliefs that the experience would never end, which were reported as being frightening. An important suggestion emerging from this work therefore is that in preparation sessions it should be discussed with patients that they may experience such thoughts and their perception of time might be affected during the infusions, though any affects are transient. Whilst individuals with psychosis/schizophrenia and family history of such disorders were excluded from the trial, it may also be worth considering specifically screening for individuals with paranoid beliefs prior to their involvement in a ketamine trial. Crucially, none of the participants reported prolonged psychosis or lasting perceptual alterations beyond the acute infusions.

Some participants described each infusion allowing further insights and understanding, likening the infusions to a three-part story. This was linked to a desire to have more than three infusions, to “go a little bit further.” Tachyphylaxis (rapidly developing tolerance) was described even over these three sessions which may suggest future doses might need to be titrated up. Indeed, similar approaches have been used in Ketamine Assisted Psychotherapy and psilocybin treatment, whereby following the first dose, subsequent doses were titrated up according to individual experiences to achieve a “mystical” (52) or “trance” state (53). Additionally, higher doses were found to be predictive of a “peak” [based on (54)] or “mystical” (35) psychedelic experience (28) which has been linked to treatment efficacy (55) and titrating up is common in the private ketamine therapeutic practise in the US (56).

All participants interviewed here reported positive experiences associated with the ketamine infusions overall despite the transient distressing experiences. This suggests that short-lived intense distress can be well-tolerated by study participants in a supportive and therapeutic setting as part of a clinical trial (38). One of the interviewed participants reported an isolated incident of recreational ketamine use following the trial. There are concerns about the abuse potential of ketamine, but this may suggest that when provided in a treatment context to participants who were appropriately screened, supervised, and followed up [see (2) for safety guidelines in human hallucinogen research] the risks are minimised.

Whilst only three of 12 participants were abstinent from alcohol at the time of interview, all participants who completed the three infusions (*N* = 11) described lasting changes in their relationship with alcohol, suggesting a shift from uncontrolled to a more controlled drinking approach: ability to remain abstinent for longer periods, reducing amount of consumption, reduction in craving to drink alcohol and changes in motives for consuming alcohol. This is a particularly valuable insight from qualitative data, which would not be possible to ascertain from the quantitative data on relapse rates alone. Similar findings of controlled drinking following treatment have also been reported in the psychedelic literature (52). This may also indicate future trials may need to consider outcomes beyond abstinence or relapse rates in evaluating ketamine's therapeutic effects for alcohol use disorders. The appropriate outcome measurements may be related to patients' motivations and expectations from the treatment, as well as perceived self-efficacy to remain abstinent, as these factors are shown to be strongly related to long term drinking outcomes (57).

There are a number of limitations to consider in the current study. Firstly, the interviews were conducted on average 2 years following participants' involvement with the KARE trial and the ketamine infusions, therefore their memory of their experiences may be subject to distortion. The acute and long-term benefits of ketamine reported by the participants here may also be explained as a result of other non-specific factors associated with taking part in a clinical trial including the rapport with the therapist, the care and attention received during the trial and wishing to please the trial team as reflected by a number of participants here [e.g., (30)]. Participants may have also felt a wish to report more positive experiences of the infusions or the trial to please the interviewers, however the fact that researchers undertaking the interviews were not previously known to the participants may have minimised this.

The current study reported patient perspectives of ketamine treatment in the context of a clinical trial for alcohol use disorders. The experiences reported here highlight the importance of supportive, safe, and a professional environment in determining individuals' acute ketamine experiences. For majority of the participants, the acute ketamine experience included dissociative effects, transient distress and perceptual distortions; though some also reported more profound religious, spiritual, and mystical experiences such as ego dissolution and epiphanies. Whilst some of these experiences including strong feelings of dissociation as well as feelings of connectedness, appeared to be related to the transformations reported by the participants, the extent to which these acute experiences contribute to ketamine's therapeutic effects for alcohol and/or substance use disorders remains to be fully investigated. The wide range of acute experiences reported here are not captured by ketamine's characterisation as a “dissociative anaesthetic.” Keeping this in mind along with issues highlighted with other terminology, a new nomenclature for describing the effects of ketamine and other “psychedelic” drugs, as well as the development of a new measure to appropriately assess ketamine's acute effects are recommended. Beyond the acute effects, potentially transformative effects of ketamine infusions and the trial are reported. For some participants, insights from the acute ketamine experience have had significant positive effects on many aspects of their lives. For the majority, the treatment is reported to have led to significant change in their relationship with alcohol. How ketamine interacts with psychotherapy, as well as the most appropriate psychotherapy to be delivered alongside ketamine treatment remains to be determined; qualitative analyses such as these are vitally important in informing such approaches.

## Data Availability Statement

Due to ethical concerns, the research data supporting this publication are not publicly available.

## Ethics Statement

The studies involving human participants were reviewed and approved by University of Exeter CLES Psychology Ethics Committee. The patients/participants provided their written informed consent to participate in this study.

## Author Contributions

OMM, CJAM, JK, and MG contributed to the conception and design of the study. OMM and JK conducted the qualitative interviews and collected survey data. OMM, CJAM, JK, KJA, and EKA were responsible for qualitative data analysis. CM was responsible for the supervision of the research study and the article. OMM led the drafting of the manuscript with JK and KJA provided critical feedback on the results section. All authors contributed to the manuscript revision, read, and approved the submitted version.

## Conflict of Interest

CJAM has consulted for Janssen Pharmaceuticals. CJAM and OMM have received research funding from Awakn Life Sciences. The remaining authors declare that the research was conducted in the absence of any commercial or financial relationships that could be construed as a potential conflict of interest.

## Publisher's Note

All claims expressed in this article are solely those of the authors and do not necessarily represent those of their affiliated organizations, or those of the publisher, the editors and the reviewers. Any product that may be evaluated in this article, or claim that may be made by its manufacturer, is not guaranteed or endorsed by the publisher.

## Section 1: Acute Experiences of the Ketamine Infusion

*1. Circumstances around deciding to participate*: Thinking back to the time period prior to starting the trial, what was it that made you consider taking part?
*2. Experiences during ketamine infusion*: Thinking back to when you were taking part in the trial, could you describe your experiences whilst being given the infusion? (Prompts—liked and disliked aspects).
  - Was the experience similar each time you were given a ketamine infusion?
  - Any more positive or negative experiences you'd like to report?
*3. Long-term experiences*: Could you now please describe your experiences in the week following the infusion?
  - Prompts: did you find that the effects of the ketamine had any lasting effects after the duration of the infusion?
  - E.g., immediately after infusion, in the days following infusion, in the weeks following infusion.
*4. Effects on world/alcohol perspective*: Do you think that the trial changed the way you see the world in any way?
  - Has anything changed around your relationship with alcohol?
  - Has anything changed with your mood?
*5. Interaction with therapy:* What were your experiences participating in the talking session [therapy/psychoeducation] following the ketamine infusion?
  - Thinking about whether the ketamine helped or did not help your engagement with the talking session

## Section 2: Ruminative Thinking Styles

In the next section, we'd like to ask you some questions about your thinking patterns, particularly about a thinking style called rumination. Have you heard of this term before? Could you tell me what your understanding of rumination is?*Rumination is a repetitive, negative, and self-critical thinking pattern about one's self, feelings, personal concerns and upsetting experiences. It is often difficult to control this thinking pattern once it starts*.

*6. Topics of rumination*: Typically, people ruminate about a great number of things. I wonder if you could tell me about some of the main things that you tend to ruminate about and your general experience of ruminating.
*7. Triggers of rumination:* Thinking about when you have these repetitive thoughts, what starts it off?
  - Prompts: negative mood, upsetting experiences?
*8. What ends the rumination:* Is there anything that tends to end/stop these thoughts?
*9. Effects of rumination on mood*: Thinking about when you're ruminating, do you notice any change in your mood or craving for alcohol?
*10. Changes in rumination*: Have you experienced any changes in your ruminative thinking following the treatment? (specify timeline: following the treatment you received during the trial).
  - Prompts: Change in how often you tend to ruminate,
  - Whether you find it easier to control ruminative thoughts
  - Change in what starts and ends rumination.

Final section involves some questions about your current alcohol and drug use.

## Section 3: Current Alcohol and Drug Use (Yes/No)

11. Have you been using alcohol since you finished participating in the trial?
  - If yes, when did you start? What do you attribute this to?
  - How frequently? (Days in month)
  - Last 14 days of alcohol use
12. Have you at used ketamine or any other drugs recreationally since finishing participation in the trial?
  - Which drugs?
  - How frequently (days in a month) what dose in a session? (A session is each occasion of use)

## Debrief

Is there anything else you would like to report related to your experience taking part in the KARE trial?Do you have any questions?
