# Supplementary material for: “This Is Something That Changed My Life”: A Qualitative Study of Patients' Experiences in a Clinical Trial of Ketamine Treatment for Alcohol Use Disorders
Source: Front Psychiatry. 2021 Aug 16;12:695335. doi: 10.3389/fpsyt.2021.695335 (PMC8415567; doi:10.3389/fpsyt.2021.695335)
Supplement: Supplementary file 1 [file Data_Sheet_1.docx]

FIRST SESSION

Now we have finished the therapy section, you are ready to receive your infusion.

We just want to take a moment to discuss what you might expect from the infusion and what you should do during it. We will start the infusion pump and then ask that you put on the headphones and listen to some soothing music. We have some suggested options but if you would rather you can choose your own music. We recommend choosing music you find calming, your mind will be active during the infusion, and something like a high-energy dance mix can cause a very unpleasant overstimulation. Many patients say instrumental music is preferable because vocals can grab your attention and distract you from the infusion experience. If you want to choose your own music pick something you’d describe with words like relaxing, tranquil, low-energy, peaceful; or something you associate with strong positive memories.

Sitting back and listening to the music will allow you to focus on your thoughts and experiences. The research nurse will ask you at 10 minute intervals to report how you are feeling and every 5 minutes we will take your blood pressure. If you have any concerns in between please let us know, for example if you feel nauseous the anaesthetist can give you something via the infusion that will very quickly make you feel better, so please tell the nurse straight away.

THE EXPERIENCE

Once the infusion begins, the ketamine will reach your brain within seconds and you’ll quickly feel its effects. You won’t be able to stand (don’t try!) or converse normally. But you won’t lose consciousness or awareness of your surroundings. Remember, the ketamine dose and rate used for these infusions is only a small fraction of what is used in surgery.

An observer will see a motionless patient who appears asleep. Physically, you will feel extremely relaxed. But your mind will be fully engaged. The sensation can be weird. But most patients describe it as pleasant.

Ketamine can make people feel somewhat spaced out, separate from their body, and can make colours and sounds change. The effects will be strongest at the start of the infusion but will get weaker as time passes. At the dose you are receiving these changes the majority of people will notice these effects. A small number of individuals may not notice the effects of ketamine at this dose.

Your experience may include something called a dissociative effect, or dissociation. Dissociation is an experience, where you partially lose awareness of your body. You can often reduce the effect simply by opening your eyes. Again, it can be weird, but most find it to be a pleasant sensation. Dissociation does not happen to every patient. Some just feel extreme relaxation.

Your mind will be very active during the infusion. In a state of deep relaxation, you may find that you’re able to reflect on past traumas or current anxieties in a very calm, matter-of-fact way – with no emotional pain. Most patients find this very satisfying, but a small percentage may find it uncomfortable. Many patients prefer to simply let their thoughts wander, without trying to steer them in any particular direction.

We would like you to try and accept your changed state, which will pass very quickly as soon as the infusion has finished.

It may help to think of these changes as a chance to see your life from a different perspective. If you are able, bring to mind the way your ideal life of abstinence would look. In order to do this you may need to bring to mind your old life and the things which you did not like about it.

If it helps you might want to make a note of a couple of reminders here that might prompt you in the session.

We find what also works well is to have a key phrase or sentence that you can repeat in your mind. Try and write down that phrase now…

The important thing is to relax and go with the experience.

If you find it too difficult to think of your abstinent life going forward then just think about the relaxation exercise you covered in the session, or sit back and enjoy the music.

The infusion will last for 40 minutes, your time perception may be altered so it may seem longer or shorter than this.

After the infusion is stopped then you will notice the effects disappearing very quickly. People who have ketamine in a therapeutic setting notice that in the hours following the infusion they may feel renewed, and they may find it easier to take a different perspective on their life. Even if the infusion effects have been very subtle, research suggests these changes can happen in your brain. We hope this experience will be useful in
