# Supplementary material for: “This Is Something That Changed My Life”: A Qualitative Study of Patients' Experiences in a Clinical Trial of Ketamine Treatment for Alcohol Use Disorders
Source: Front Psychiatry. 2021 Aug 16;12:695335. doi: 10.3389/fpsyt.2021.695335 (PMC8415567; doi:10.3389/fpsyt.2021.695335)
Supplement: Supplementary file 2 [file Data_Sheet_2.docx]

SECOND AND THIRD SESSION

Now we have come to the end of the therapy section, you are ready to receive your next infusion.

We just want to take a moment to review what you might expect from the infusion and what you should do during it. Again, after we start the infusion pump and then ask that you put on the headphones and listen to some soothing music. This will allow you to focus on your thoughts and experiences. The research nurse will ask you at 10 minute intervals to report how you are feeling and every 5 minutes we will take your blood pressure. If you have any concerns in between please let us know, for example if you feel nauseous the anaesthetist can give you something that will very quickly make you feel better, so please tell the nurse straight away.

THE EXPERIENCE

The effects of ketamine will be strongest at the start of the infusion but will get weaker as time passes. At the dose you are receiving these changes will be quite mild but for the majority of people they will be noticeable. A small number of individuals may not notice the effects of ketamine at this dose, so it is important you all do the following exercise anyway.

We would like you to try and accept the changed state of seeing the world, which will pass very quickly as soon as the infusion has finished.

Think of these changes, if you experience them, as a chance to see your life from a slightly different perspective. If you are able, bring to mind the way your ideal life of abstinence would look. In order to do this you may need to bring to mind your old life and aspects which you did not like about it.

If it helped you before, then again note down some reminders, or a key phrase to think of during the session:

Again please try to relax and go with the experience.

If you find it too difficult to think of your abstinent life going forward then just think about the relaxation exercise you covered in the session, or sit back and enjoy the music.

The infusion will last for 40 minutes, your time perception may be altered so it may seem longer or shorter than this.

After the infusion is stopped then you will notice the effects disappearing very quickly. People who have ketamine in a therapeutic setting notice that in the hours following the infusion they may feel renewed or changed, and they may find it easier to take a different perspective on their life.
